# Growth conditions and environmental factors impact aerosolization but not virulence of *Francisella tularensis* infection in mice

**DOI:** 10.3389/fcimb.2012.00126

**Published:** 2012-10-11

**Authors:** Seth A. Faith, Le'Kneitah P. Smith, Angela S. Swatland, Douglas S. Reed

**Affiliations:** Regional Biocontainment Laboratory, Center for Vaccine Research, University of PittsburghPittsburgh, PA, USA

**Keywords:** tularemia, *Francisella tularensis*, respiratory infection, aerosol exposure, mice

## Abstract

In refining methodology to develop a mouse model for inhalation of *Francisella tularensis*, it was noted that both relative humidity and growth media impacted the aerosol concentration of the live vaccine strain (LVS) of *F. tularensis*. A relative humidity of less than 55% had a negative impact on the spray factor, the ratio between the concentration of LVS in the aerosol and the nebulizer. The spray factor was significantly higher for LVS grown in brain heart infusion (BHI) broth than LVS grown in Mueller–Hinton broth (MHb) or Chamberlain's chemically defined medium (CCDM). The variability between aerosol exposures was also considerably less with BHI. LVS grown in BHI survived desiccation far longer than MHb-grown or CCDM-grown LVS (~70% at 20 min for BHI compared to <50% for MHb and CCDM). Removal of the capsule by hypertonic treatment impacted the spray factor for CCDM-grown LVS or MHb-grown LVS but not BHI-grown LVS, suggesting the choice of culture media altered the adherence of the capsule to the cell membrane. The choice of growth media did not impact the LD_50_ of LVS but the LD_99_ of BHI-grown LVS was 1 log lower than that for MHb-grown LVS or CCDM-grown LVS. Splenomegaly was prominent in mice that succumbed to MHb- and BHI-grown LVS but not CCDM-grown LVS. Environmental factors and growth conditions should be evaluated when developing new animal models for aerosol infection, particularly for vegetative bacterial pathogens.

## Introduction

Tularemia is a disease found throughout North America, Europe, and Asia that can be contracted by contact, arthropod vectors, or inhalation (Dennis et al., 2001; Pechous et al., 2009). The causative agent, *Francisella tularensis*, was first identified in 1912. *F. tularensis* is a gram negative non-motile coccobacillus and is considered a facultative intracellular bacterium. *F. tularensis* can infect a significant number of mammals, arthropods, and even fresh-water amoebae. In the environment *F. tularensis* has been isolated from water, soil, and various wild animals, especially rabbits, hares, and rodents. In mammals the primary cell targets are thought to be macrophages and dendritic cells. Four subspecies have been identified: tularensis, holarctica, mediasiactica, and novicida (Ellis et al., 2002). Subspecies tularensis strains, also designated as type A, are the most virulent and are most frequently found in North America (type A can be further divided into A.1 and A.2) (Gurycova, 1998; Staples et al., 2006). Subspecies holarctica, or type B strains, are not as virulent as type A but they can cause human disease and are found throughout North America and Europe.

*F. tularensis* was evaluated as a biological weapon and weaponized by both the former Soviet Union and the United States of America (the USA renounced the use of offensive biological weapons in 1969) prior to ratification of the Biological Weapons Convention (BWC) Treaty. In addition to being infectious when inhaled, *F. tularensis* could be easily grown to high titers and caused significant morbidity and mortality at very low doses (Dennis et al., 2001). A World Health Organization (WHO) report concluded that an aerosol release of 50 kg of *F. tularensis* over a metropolitan area of 5 million would result in 250,000 casualties and 19,000 deaths (Dennis et al., 2001).

Because of the virulence and high infectivity of *F. tularensis*, attenuated strains are commonly used in laboratories that lack the specialized containment required. Subspecies novicida, which can cause lethal disease in mice but do not typically cause disease in humans, is often used (Pechous et al., 2009). However, novicida strains do not express a capsule which is required for the virulence of type A and type B strains (Cox and Goldberg, 1972; Golovliov et al., 2003; Jia et al., 2010; Bandara et al., 2011). LVS, a classically attenuated type B (holarctica) strain, is avirulent in humans, rabbits, rats, and nonhuman primates (NHP) but can cause lethal disease in mice (Lyons and Wu, 2007). Unlike novicida strains, LVS expresses a capsule which is thought to be similar to that of the more virulent strains. An LVS mutant which did not express capsule was found to be able to replicate inside macrophages but was otherwise attenuated in mice (Golovliov et al., 2003). Because of the extraordinarily low dose of virulent *F. tularensis* type A required to kill mice when given by i.n. inoculation or inhalation of small particle aerosols, it has been argued that LVS infection of mice is a good model of the human disease and useful for studying protective innate and adaptive immune responses, particularly in laboratories that are not registered for the use of select agents (Elkins et al., 2003).

Well-characterized animal models are needed to meet the requirements of the FDA's Animal Rule for licensure of medical countermeasures (2002). The Animal Rule allows the use of efficacy studies in animals to support licensure in lieu of human efficacy. As part of meeting the requirements laid out in the Animal Rule, additional guidance from the FDA stipulates that the route of exposure must be the same as the anticipated route of human exposure and that reliable quantification and reproducibility of the challenge dose should be demonstrated (FDA, 2009). To achieve the goal of a standardized, reproducible system for exposing animals, the impact of environmental factors needs to be assessed. We report here our efforts to establish a system for reproducibly infecting animals with *F. tularensis* and infect mice with small particle aerosols containing LVS as a surrogate model of human disease.

## Materials and methods

### Bacteria

Frozen stocks of *F. tularensis* subsp. *holarctica*, attenuated live vaccine strain (LVS) (graciously provided by Dr. Gerald J. Nau, University of Pittsburgh) and subsp. tularensis strain SCHU S4 (BEI Resources, Manassas, VA) were plated onto cysteine heart agar supplemented with 1% bovine hemoglobin (CHAH; Becton-Dickinson, Franklin Lakes, NJ) and incubated at 37°C with 5% CO2 for 48 h. Isolated colonies were resuspended in phosphate buffered saline (PBS, pH 7.2) to obtain a bacterial suspension with optical density at 600 nm (OD_600_) = 0.100 ± 0.020. Five-hundred microliters of bacterial suspension was added to 25 ml of broth culture and incubated at 37°C in vented 125 ml baffled flasks with agitation at 250 RPM. Bacteria were enumerated by diluting samples 10-fold in PBS and plating onto CHAH. Colony forming units (cfu) were assessed between 48–96 h incubation. All work with SCHU S4 was conducted at biosafety level (BSL)-3 in the University of Pittsburgh Regional Biocontainment Laboratory. The University of Pittsburgh Regional Biocontainment Laboratory is a Registered Entity with the CDC/USDA for work with *F. tularensis*.

### Broth media

Three different broth media were evaluated in these studies: (1) modified Mueller–Hinton medium (MHb) supplemented with 1.23 mM calcium chloride dihydrate, 1.03 mM magnesium chloride hexahydrate, 0.1% (wt/vol) glucose, 0.025% (wt/vol) ferric pyrophosphate, and 2% (vol/vol) IsoVitaleX (Becton-Dickinson, Franklin Lakes, NJ); (2) Chamberlain's chemically defined medium (CCDM) (Chamberlain, 1965); or (3) brain heart infusion (BHI) broth (Becton-Dickinson, Franklin Lakes, NJ). MHb was prepared and sterilized by autoclaving in accordance with manufacturer's instructions. BHI and CCDM were sterilized by membrane filtration after pH was confirmed.

### Mice

Eight to ten week old female Balb/c mice (Charles River, MA) were provided with food and water *ad libitum* and housed in bio-containment caging (Allentown Inc, Allentown, NJ). All animal work was performed according to Institutional Animal Care and Use Committee (IACUC) guidelines of The University of Pittsburgh.

### Clinical scoring and euthanasia

Mice were weighed daily and monitored at least twice daily for changes appearance and behavior; mice that were determined to be moribund (either by score or greater than 20% weight loss) were humanely euthanized promptly by carbon dioxide intoxication and necropsied after death was confirmed. Scoring was given as a combined score of natural behavior, provoked behavior, and appearance. Natural behavior: normal = 1; less peer interaction = 2; little peer interaction, less mobile = 3; no peer interaction, vocalization, restless or still = 4; Provoked behavior: normal = 1; subdued but normal when stimulated = 2; subdued even when stimulated = 3; unresponsive even when stimulated = 4; Appearance: normal = 1; reduced grooming = 2; dull/rough coat or ocular/mucosal discharge = 3; hunched, piloerection = 4.

### Aerosol exposures

Aerosols of LVS cultures were generated using 3-jet Collison nebulizers (BGI, Inc. Waltham, MA) that create a monodisperse aerosol approximately 1–2 μm in size controlled by the AeroMP bioaerosol exposure system (Biaera Technologies, Hagerstown, MD). Mice were exposed inside either whole-body (Biaera Technologies) or nose-only (CH Technologies, Westwood, NJ) rodent exposure chambers. Aerosol sampling was performed using all-glass impingers (AGI-30; Ace Glass, Vineland, NJ) containing 10 ml of culture media and 0.08% Antifoam A (Sigma-Aldrich, St. Louis, MO). Nebulizer and AGI contents were diluted 10-fold in PBS and plated onto CHAH plates to quantify recovered bacteria. Aerosol concentration was determined as the product of the AGI concentration and the volume of liquid in the AGI divided by the product of the AGI flow rate (6 l/min) and duration of the aerosol (Roy and Pitt, 2005). Spray factor (SF) was calculated as the aerosol concentration divided by the nebulizer concentration. Respiratory minute volume was determined using Guyton's formula (Guyton, 1947). Presented dose was measured as the product of the minute volume by the aerosol concentration and the duration of the aerosol (Roy and Pitt, 2005).

### Capsule removal

Adapted from (Hood, 1977), *F. tularensis* LVS was grown in CCDM, MHb, or BHI to pre-stationary phase, resuspended in 0.8 or 10% NaCl solution (pH 6.4–6.8) and stored 4 days at 4°C. Bacteria were centrifuged 2000g × 30 min and cell pellets were resuspended in respective media prior to aerosolization.

### Desiccation assay

*F. tularensis* LVS was grown in CCDM, MHb, or BHI to pre-stationary phase, washed with PBS and resuspended in PBS. Sterile cellulose nitrate gridded filter papers (Whatman, Piscataway, NJ) were placed in sterile petri dishes and 100–300 cfus of washed *F. tularensis* were instilled onto each paper. At the reported time intervals from 0 to 60 min postinstillation, papers were placed inoculated side up onto CHAH plates and incubated at 37°C with 5% CO_2_ for 48–96 h. Percent survival was calculated against non-desiccated LVS inoculums.

### Tissue colonization

Lung, liver, and spleen organs were removed from moribund and euthanized mice, weighed and frozen at −80°C. Thawed organs were homogenized in PBS with an Omni TH homogenizer with disposable plastic soft tissue probes (Omni International, Kennesaw, GA), filtered through a 0.75 micron mesh (Fisher Scientific), diluted 10-fold in PBS and plated on CHAH plates for enumeration of bacterial load. The quantity of bacteria per sample was calculated as cfu per gram of tissue.

### Statistics

Significance tests were performed with Graph Pad Prism Software (GraphPad, La Holla, CA) using unpaired Student's *t*-tests and One-Way ANOVA. Probit slope analysis of survival curves was used to determine the LD_50_ using NCSS 2004 software (Number Cruncher Statistical Systems, Kaysville, Utah).

## Results

**Figure 1:**
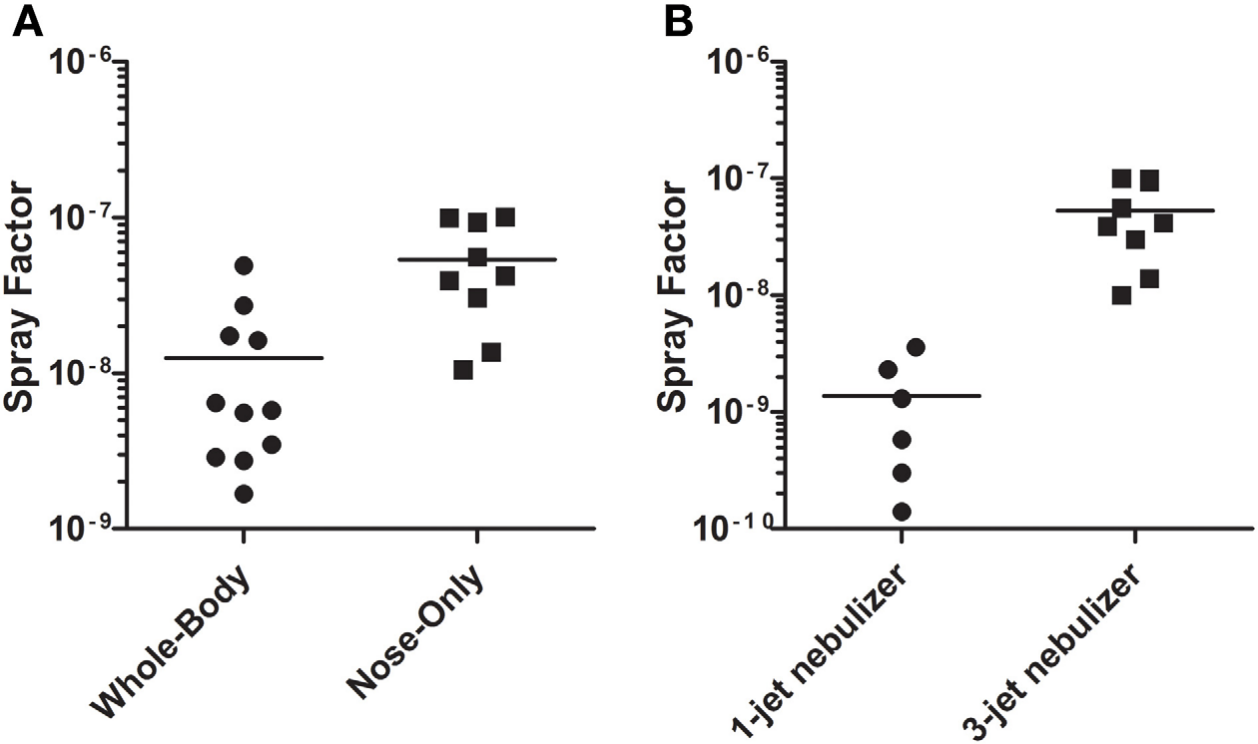
**Spray factor of LVS with different exposure chambers and nebulizers.** *F. tularensis* strain LVS was grown in MHb overnight and then aerosolized into a whole-body or nose-only exposure chamber **(A)** or with a Collison nebulizer using a 1- or 3-jet nozzle into a nose-only exposure chamber **(B)**. Aerosol samples were collected in an AGI and plated to determine aerosol concentration of LVS. Plots show the spray factor (ratio of aerosol concentration to nebulizer concentration) for individual aerosol exposures (circles and squares) and the median (line) value.

As part of initial efforts to develop a mouse model for pneumonic tularemia, aerosols were conducted without animals to define factors important for achieving consistent, reproducible dosing. Spray factor is the ratio of the aerosol concentration to the nebulizer concentration; it can be used as both a means to determine the nebulizer concentration needed to achieve a desired presented dose and the performance of an aerosol relative to other aerosols of the same agent (Roy and Pitt, 2005). The higher the spray factor, the lower the concentration needed in the nebulizer to achieve a desired presented dose. Initial aerosols to determine the spray factor of *F. tularensis* were conducted using LVS grown in Mueller–Hinton broth (MHb) overnight at 37°C in a shaker after a two day culture at 37°C, 5% CO_2_ on CHAH. As shown in Figure 1A, when using a 3-jet Collison nebulizer the spray factor for LVS was significantly higher with a nose-only exposure chamber than with a whole-body exposure chamber (5.4 × 10^−8^ vs. 1.3 × 10^−8^, respectively; *p* = 0.0087). Using the nose-only chamber, the spray factor for 1-jet Collison (1.4 × 10^−9^) was considerably lower than had been found with the 3-jet Collison (Figure 1B); the difference was statistically significant (*p* = 0.0023).

**Figure 2:**
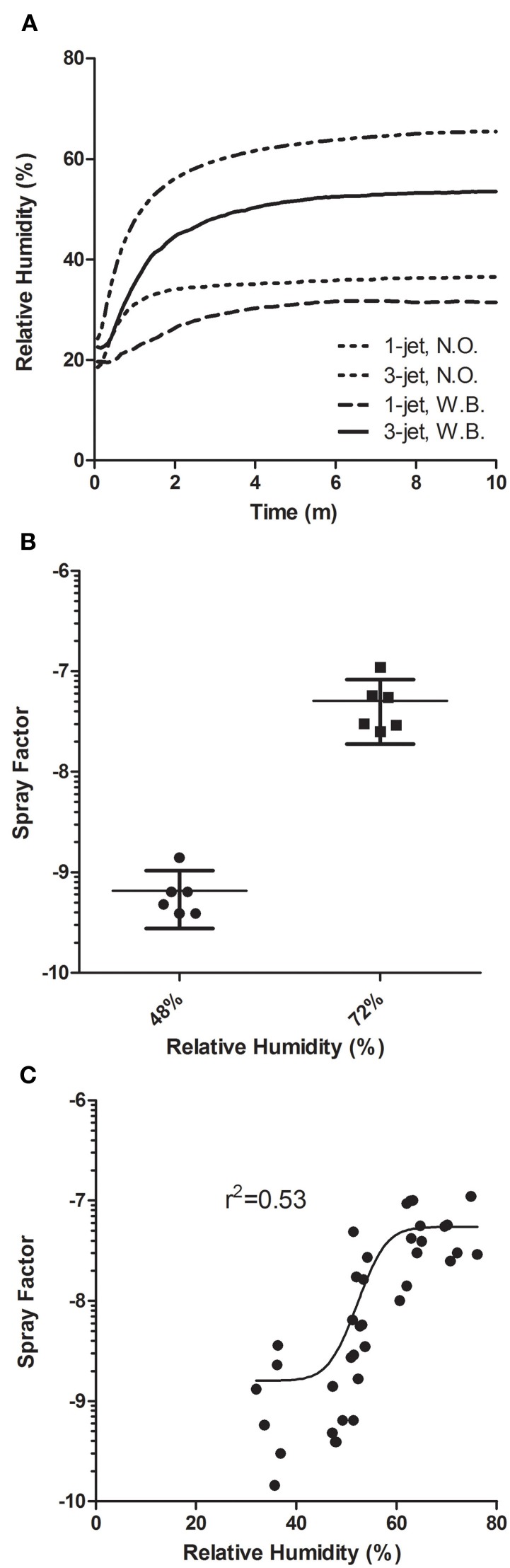
**Effect of relative humidity on aerosolization of LVS.** Relative humidity during exposures is monitored and logged by the AeroMP system. In **(A)**, the graph shows the change in relative humidity over time using different nebulizer/exposure chamber combinations without using the humidification loop to artificially increase humidity. In **(B)**, the graph shows spray factors for individual exposures (squares and triangles) with the median and standard deviations at two different relative humidity levels. In **(C)** the graph shows the spray factors for a number of runs at a range of relative humidity values. Data in **(C)** were analyzed in Prism using four-parameter logistic regression.

Prior reports have shown that for vegetative bacteria in general and for *F. tularensis* specifically, relative humidity levels inside the exposure chamber can impact the aerosol concentration, thereby changing the spray factor (Cox and Goldberg, 1972). The Biaera AeroMP control system monitors temperature, humidity, and pressure in the exposure chamber throughout each exposure and saves that information in a log file. Figure 2A shows the relative humidity recorded for the nose-only and whole-body chambers using either 1-jet or 3-jet Collison nebulizers. Although they all roughly started at about the same level, considerably higher relative humidity was achieved with the 3-jet nebulizer, particularly in the nose-only exposure chamber. A humidification chamber was used to compare the spray factor for LVS using a 3-jet Collison nebulizer in a whole-body chamber. Increasing the average relative humidity to 72% led to a 2-log increase in spray factor over unmodified chamber air which had a average relative humidity of 48% (Figure 2B). The difference in spray factor (5.1 × 10^−8^ vs. 6.6 × 10^−10^ for 72 and 48% RH, respectively) was statistically significant (*p* = 0.01). A series of aerosols conducted at different RH found a dramatic improvement in spray factor between 50–60% RH for LVS (Figure 2C).

**Figure 3:**
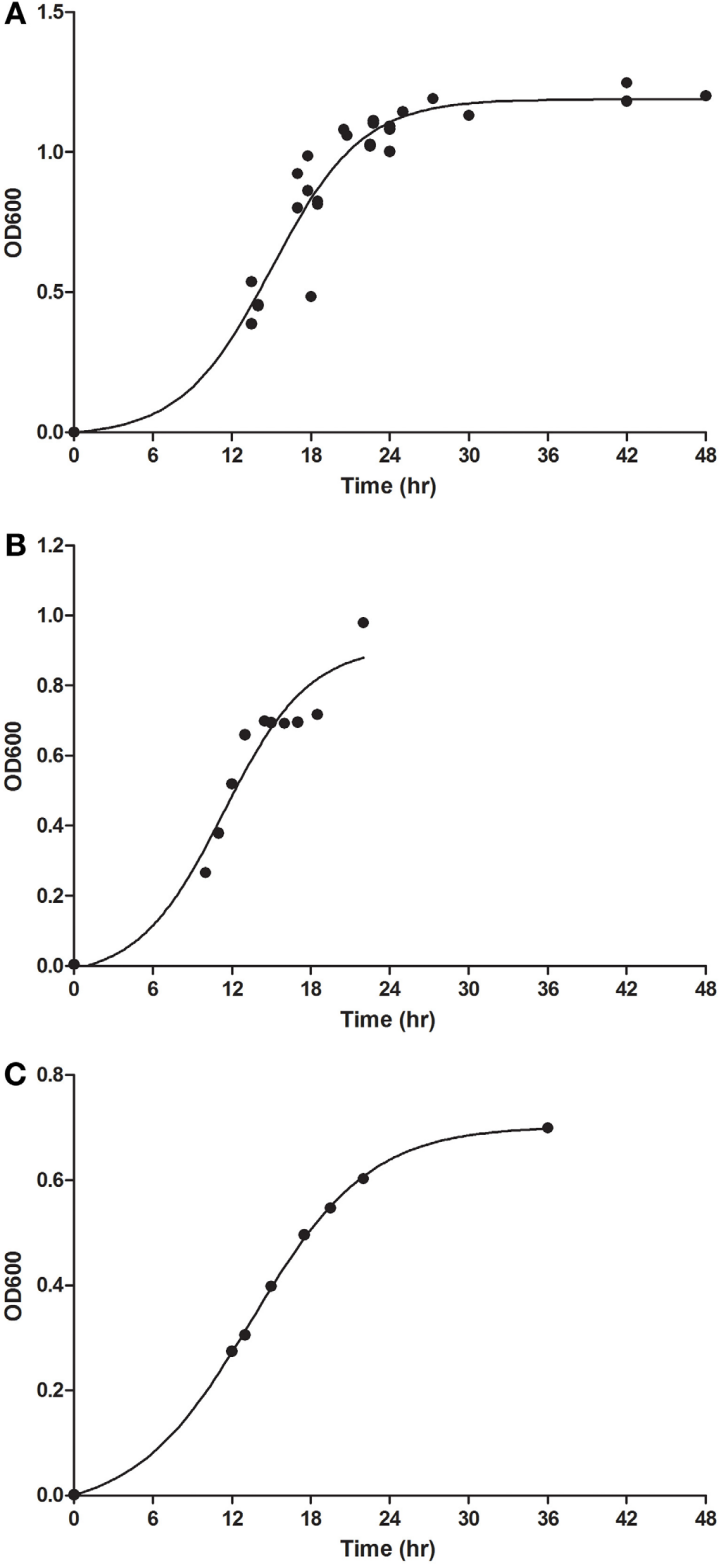
**Growth of LVS in different liquid broth media.** LVS was cultured for 2 days on CHAH prior to being cultured overnight in **(A)** MHb, **(B)** CCDM, and **(C)** BHI liquid culture media. Graphs show the change in optical density at 600 nm at different times after the culture was started (circles). Boltzmann sigmoidal semi-logistic regression was used to fit the line shown on each graph.

Another potential factor in aerosolization of *F. tularensis* was the growth media used. The method adopted was modified from a colleague at USAMRIID, Dave Waag, which itself was a modified protocol from that used to grow and prepare *Yersinia pestis* for aerosolization (Davis et al., 1996). Initial aerosols had used LVS grown in MHb; however, a review of the literature identified CCDM and BHI as other liquid media that could be used to culture of LVS (Chamberlain, 1965; Hazlett et al., 2008). LVS was grown in all three culture media and samples were taken at a number of points to read the OD_600_ and determine bacterial concentration (Figure 3). Although the OD_600_ values obtained at given timepoints were different between the three culture media, regression analysis indicates that the time at which the peak of the curve occurred was approximately the same. For aerosolization experiments, 18 h was chosen for all three culture conditions as this corresponded with late-log/pre-stationary phase growth.

**Figure 4:**
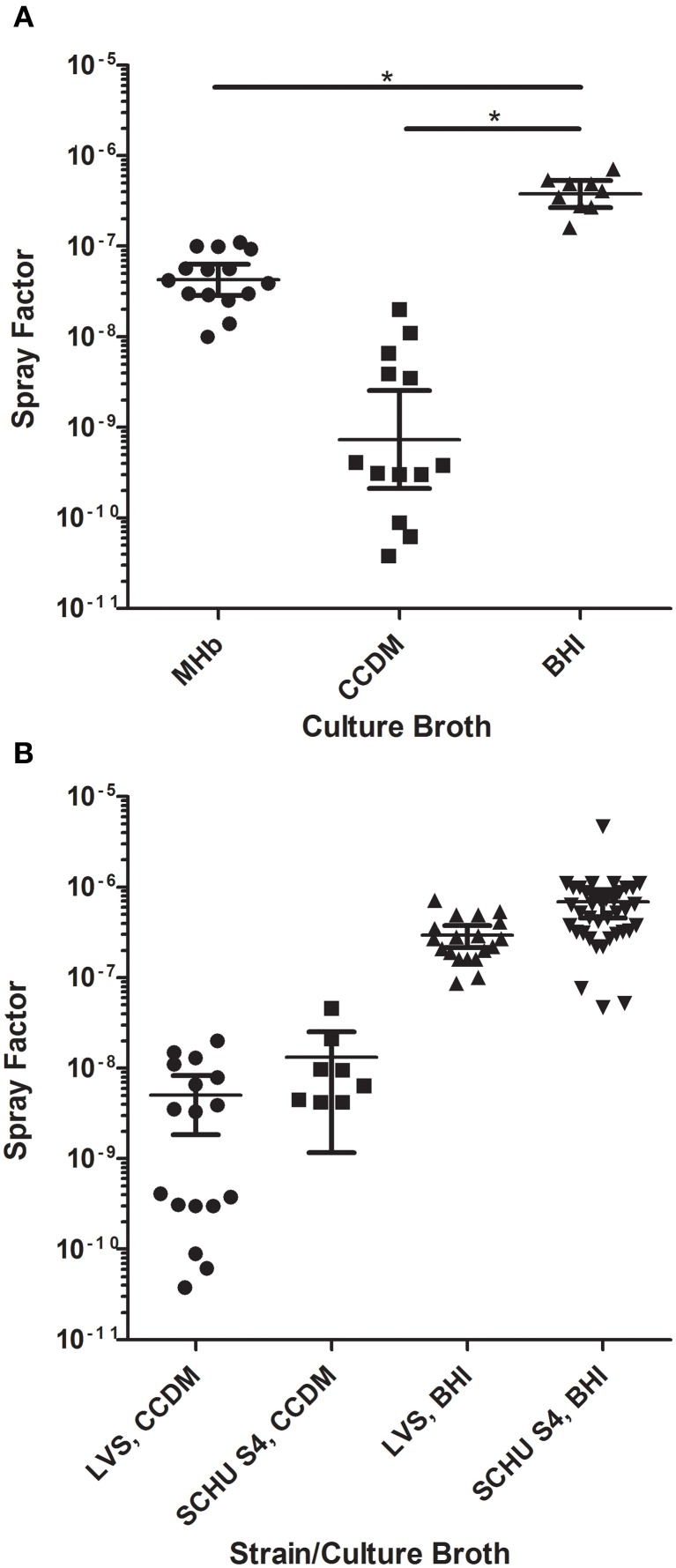
**Impact of growth media on aerosolization of *F. tularensis*.** Graphs show spray factor for *F. tularensis* after growth in different liquid culture media. **(A)** Shows the spray factor for individual aerosol exposures of LVS grown in MHb (circles), CCDM (squares), and BHI (triangles) along with the mean and standard deviation for each growth condition. Lines with (*) are statistically significant based on two-tailed Student's *t*-test. **(B)** Spray factors for individual aerosol exposures of LVS or SCHU S4 grown in CCDM or BHI along with mean and standard deviation for each growth condition.

LVS was then grown in all three culture conditions using the established protocol (2 days on CHAH agar; 18 h in broth media, shaking at 37°C), harvested, and aerosolized over a range of nebulizer concentrations. Fresh culture media of the same type used to grow LVS was used for collection of aerosol samples. Relative humidity was kept high (75%) to ensure differences in humidity did not alter the spray factor. The results are shown in Figure 4A. LVS grown in BHI gave the best overall spray factor (2.9 × 10^−7^) while MHb was approximately 1 log lower (3.6 × 10^−8^) and CCDM-grown LVS was the lowest (4.7 × 10^−9^). Using a One-Way ANOVA, the spray factor for BHI was significantly better than that obtained for both MHb and CCDM (*p* < 0.05); the spray factor for MHb was not, however, significantly different from CCDM. The coefficient of variation for BHI (0.56) was also lower than that obtained for either MHb (0.85) or CCDM (1.15). The effects of culture broth on spray factor were not exclusive to LVS; similar data were obtained with a virulent type A strain, SCHU S4 (Figure 4B). Differences in spray factor between LVS and SCHU S4 have been reported (Brasel et al., 2009) but using our methods for culturing and aerosolizing *F. tularensis* no significant difference in spray factor was seen between LVS or SCHU S4 in either CCDM or BHI.

**Figure 5:**
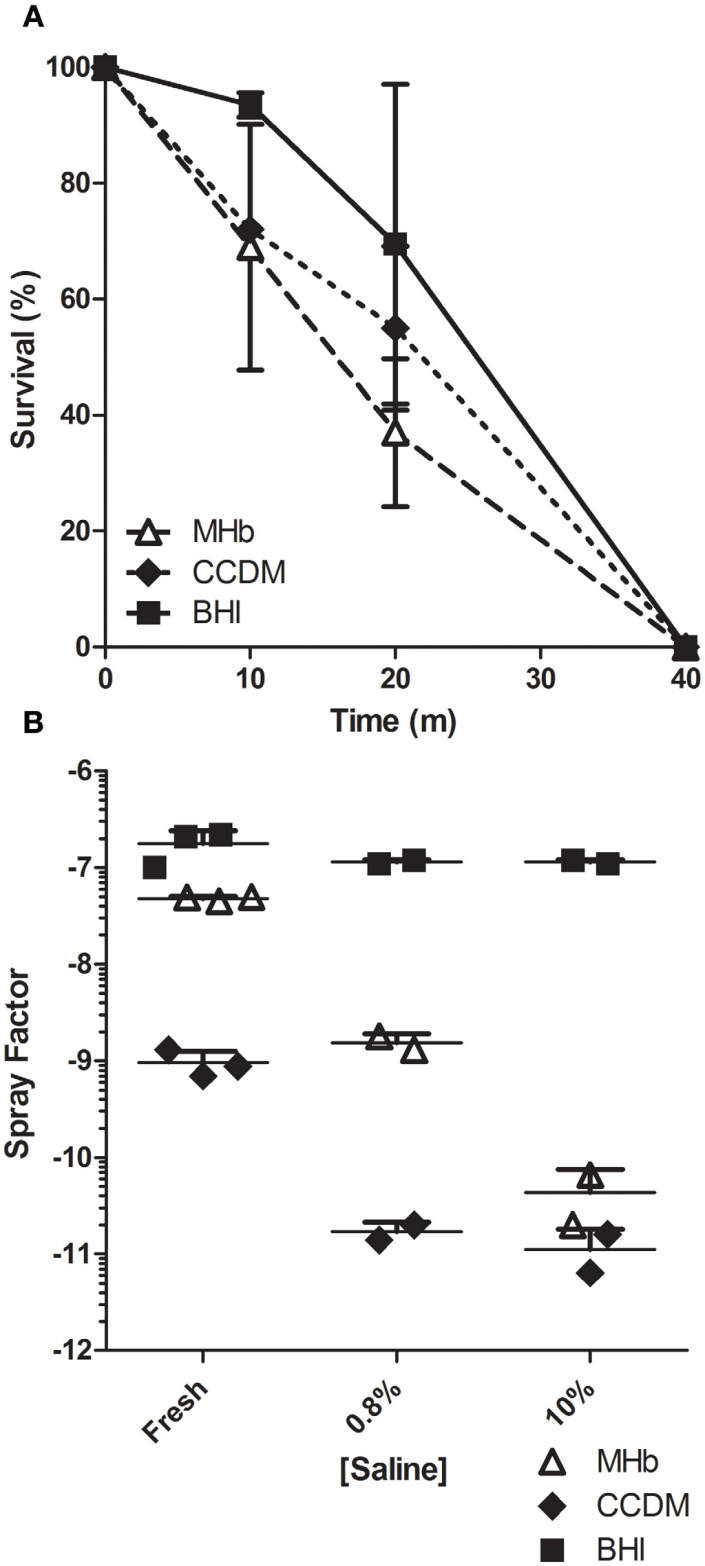
**Growth media impact on LVS desiccation survival time.** Graph in **(A)** shows impact of liquid broth culture on survival time of LVS from desiccation on Whatman filter paper. Data shown is mean values for two experiments with the standard deviation. Graph in **(B)** shows spray factor for individual aerosol exposures and means of LVS cultures that were washed and incubated for 3 days in fresh media, 0.8 or 10% saline.

In a prior report establishing the importance of RH in aerosolization of *F. tularensis*, it was found that removing the capsule that protects the bacterium rendered it susceptible to increased salt concentration (Hood, 1977). The spray factors obtained from aerosolization of LVS grown in the three culture broths suggested that capsule production was different between the three culture broths and could explain the aerosol results. To evaluate whether this was the case, LVS was grown in each broth condition overnight and evaluated for how long it could survive desiccation on Whatman filter paper (Figure 5A). At 10 and 20 min after placement on the filter paper, survival was better for BHI-grown LVS than for CCDM or MHb grown LVS. Only at 10 min was this difference statistically significant and only in comparison to CCDM (*p* = 0.006). Regardless of culture condition, LVS did not survive at 40 min of desiccation.

Another possible explanation for the aerosol data was that culturing in the different broth media induced differences in the anchoring of the capsule to the cell membrane of *F. tularensis*. Incubation in 10% saline for 3 days at 4°C has been shown to be sufficient to remove the capsule from *F. tularensis*, thereby rendering the bacterium avirulent in guinea pigs (Hood, 1977). LVS grown in the three culture broths was washed twice to remove any remaining broth and incubated for three days at 4°C in either 10% saline, physiological saline (0.85%), or in fresh culture media. Following the 3 days treatment, each was washed twice, resuspended in fresh culture media, and aerosolized. The spray factors resulting from that experiment are shown in Figure 5B. As expected, incubation of CCDM or MHb grown LVS in saline resulted in a considerable decrease in spray factor. Capsule was most easily stripped from CCDM-grown LVS as illustrated by the difference in spray factor at 0.85% saline, which was the same as incubation at 10% saline. LVS grown in MHb appeared more resistant, with an intermediate loss in spray factor at 0.85% saline although the overall decline in spray factor at 10% saline compared to fresh media was higher (3 log_10_ vs. 2 log_10_ for MHb and CCDM, respectively). The spray factor for both was virtually identical after incubation in 10% saline, suggesting that at this concentration of saline the capsule was completely removed and offered no protection against saline in the aerosol. BHI grown LVS, in contrast, was completely resistant to stripping of the capsule; at either 0.85 or 10% saline there was no difference in the spray factor compared to incubation in fresh culture media.

**Figure 6:**
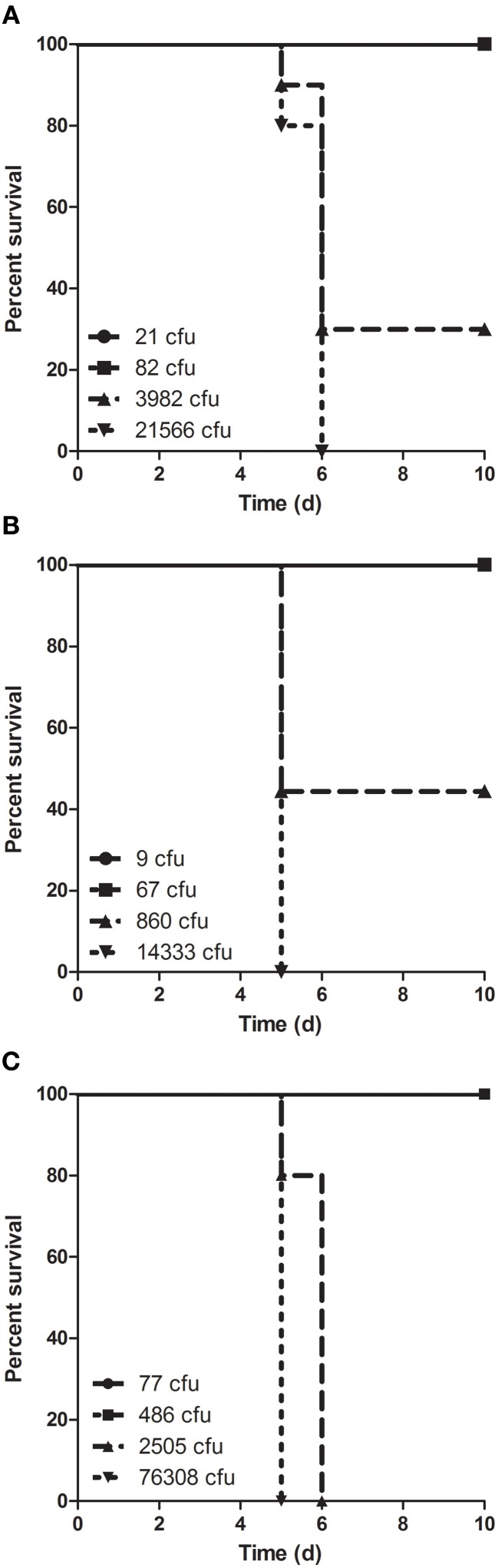
**Survival of mice after aerosol exposure to LVS grown in different culture media.** Mice were exposed to aerosols containing LVS grown in **(A)** MHb, **(B)** CCDM, and **(C)** BHI at a range of doses sufficient for determining the median lethal dose (*n* = 10 mice per dose). Graphs are Kaplan–Meier plots with doses shown in lower left.

Balb/c mice were exposed to small particle aerosols containing LVS at a range of doses to evaluate the virulence of aerosolized *F. tularensis* grown in the different culture conditions (Figure 6). Mice were not allowed to proceed to death but were euthanized when they met either a series of clinical signs indicative of a moribund state or greater than 20% weight loss. Regardless of dose, mice were found to be moribund between 5–6 days after exposure. At lower doses, there was little difference in survival between the different culture conditions but at higher doses there was the suggestion that BHI-grown LVS might be more virulent. Probit analysis confirmed that the LD_50_ was virtually identical between the three culture conditions and that the time of death was also essentially the same (Table 1). The probit analysis also indicated that the LD_99_, the dose at which one would expect the death of all unprotected mice, was almost a half-log_10_ lower with BHI than with either CCDM or MHb.

**Table 1:** **Impact of culture media on virulence of LVS**.

	**MHb**	**CCDM**	**BHI**
LD_50_ (cfu)	1210	950	1280
LD_99_ (cfu)	1.5 × 10^5^	1.2 × 10^5^	4.4 × 10^4^
MTD^*^	5.2	5.8	5.5

* Mean time to death.

**Figure 7:**
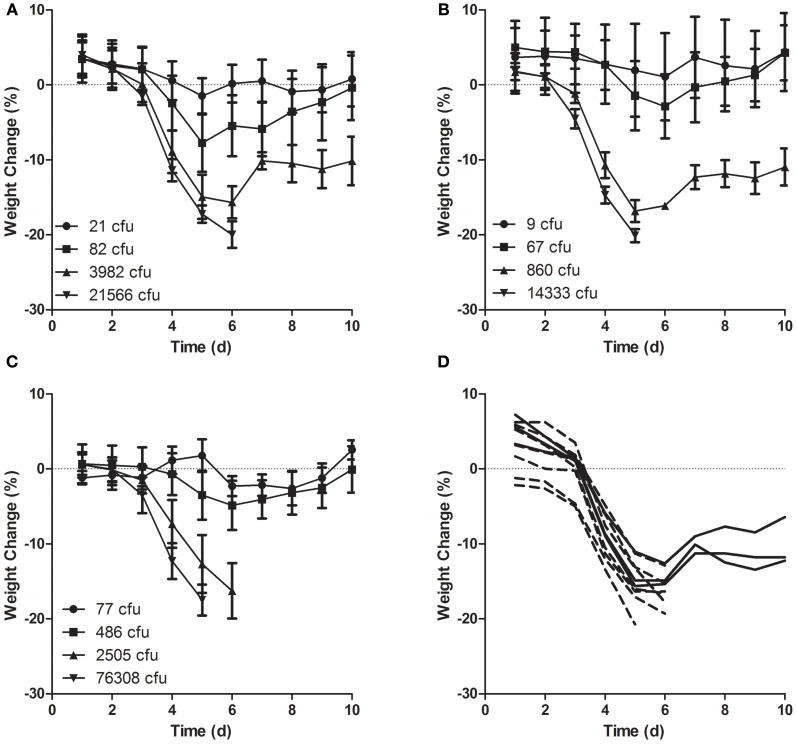
**Weight loss in mice after aerosol exposure to LVS grown in different culture media.** Mice were weighed daily beginning the day of exposure and continuing through day 10 after infection. Mice were exposed to aerosols containing LVS grown in **(A,D)** MHb, **(B)** CCDM, and **(C)** BHI at a range of doses (*n* = 10 per dose). Graphs **(A–C)** show average daily weight lost from baseline (day 0) for each group; error bars show standard deviation; graph **(D)** shows individual weights for mice in second highest dose group (3982 cfu) exposed to LVS grown in MHb.

Mice were also weighed daily to assess illness by weight loss (Figure 7). Regardless of culture condition, little change in weight was seen at the lowest challenge doses where all mice survived. At higher doses, weight loss was first notable on either day 3 or 4 after exposure and declined dramatically through day 6. Linear regression analysis indicated that at the highest doses, mice infected with LVS grown in MHb or CCDM lost weight at a faster rate than did mice infected with LVS grown in BHI although the weight loss rate was also dose dependent and did not predict survival. As shown in Figure 7D, mice that survived exposure at a high dose of MHb grown LVS lost weight at a rate equivalent to that of mice that succumbed in the same group.

**Figure 8:**
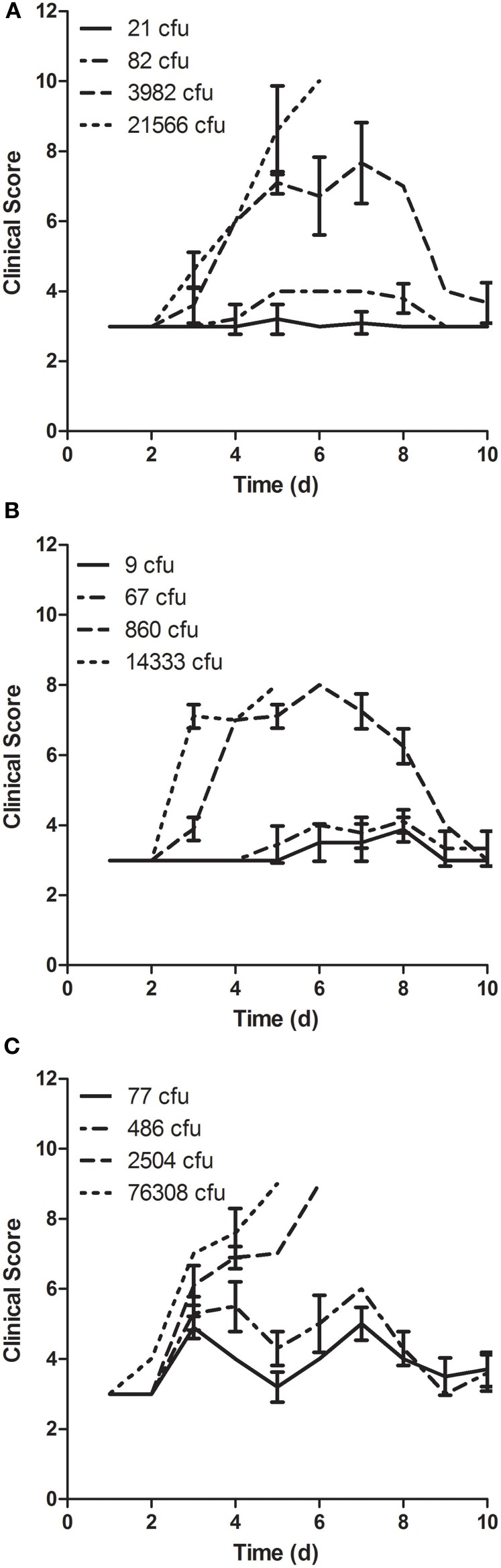
**Clinical scoring of disease in mice after aerosol exposure to LVS grown in different culture media.** Mice were scored daily beginning the day of exposure and continuing through day 10 after infection for changes in behavior and appearance. Mice were exposed to aerosols containing LVS grown in **(A)** MHb, **(B)** CCDM, and **(C)** BHI at a range of doses (*n* = 10 per dose). Graphs show average daily score for each group; error bars show standard deviation.

In addition to weight loss, mice were scored for changes in behavior and appearance (Figure 8). Clinical signs noted included dull coat, piloerection, hunching, rapid breathing, less peer interaction, and response to gentle prodding. Regardless of culture condition, at higher doses the onset of clinical signs coincided or slightly preceded detectable weight loss. Clinical signs were noted in mice that survived infection, including those that demonstrated little if any weight loss. There were differences in the onset and severity of clinical signs noted in mice that survived infection with LVS grown in different culture broth, with both earlier onset and greater severity after infection with LVS grown in BHI (see clinical scores for the mice infected with lower doses in Figure 8).

**Figure 9:**
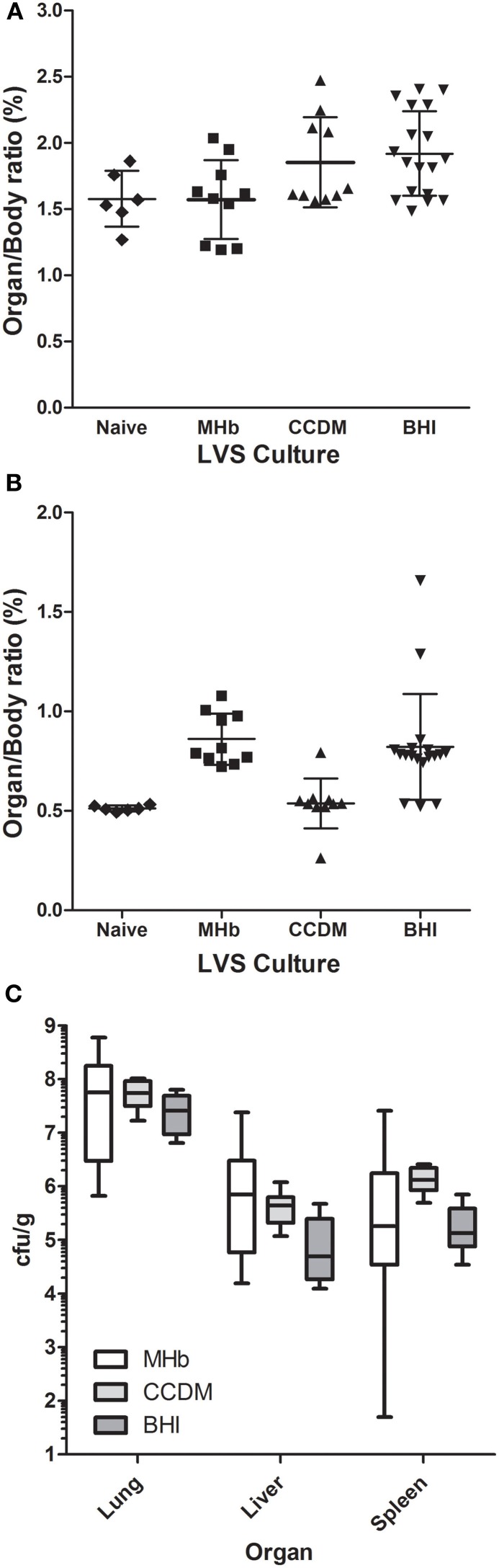
**Bacterial load in tissues of mice after aerosol exposure to LVS grown in different culture media.** Mice became moribund after LVS infection were euthanized and necropsied. The liver, lung, and spleen were removed, weighed, and then analyzed to determine the bacterial titer in each tissue. Graphs in **(A,B)** show weight for lungs **(A)** and spleens **(B)** at time of necropsy from individual mice infected with LVS grown in MHb, CCDM, or BHI as well as the mean for each group and standard deviation. Weights of tissues from naive, uninfected mice are also shown. Graph in **(C)** shows a box and whiskers plot for the bacterial titer from the lung, liver, and spleen of mice that succumbed to infection with LVS grown in MHb (white boxes), CCDM (light gray boxes), or BHI (dark gray boxes).

Mice that succumbed to LVS were necropsied after euthanasia. Lungs and spleens were weighed and bacterial load was assessed in lung, liver, and spleen (Figure 9). The ratio of lung weight to body weight after infection with LVS was roughly equivalent for all three culture conditions to that of the lungs from normal mice. However, only for BHI-grown LVS was the difference significant from naïve mice (*p* = 0.03). For the spleen, the organ/body weight ratio was higher than normal from mice infected with LVS grown in MHb or BHI (*p* < 0.001). In mice infected with LVS grown in CCDM, there was no difference in spleen weight relative to controls. LVS titers in each of the organs examined were far more variable in mice infected with MHb-grown LVS than either CCDM- or BHI-grown LVS. LVS titers were highest in the lung and levels were roughly equivalent across all three culture conditions. In the liver, LVS titers were a log higher for MHb and CCDM grown LVS than BHI grown LVS, although the difference was only significant when comparing BHI and CCDM (*p* = 0.009). In the spleen, the concentration of LVS was a log higher in the spleens of mice infected with CCDM grown LVS than either MHb or BHI, although the difference was again only significant with the BHI-grown LVS (*p* = 0.0005).

## Discussion

To meet the requirements of the FDA's Animal Rule requires careful characterization and standardization of experimental aerosols with the pathogen of interest to achieve reproducible infection at desired challenge doses. A survey of the published literature and discussions with other laboratories performing research on *F. tularensis*, revealed that a variety of methods were used to culture and aerosolize *F. tularensis*. Some laboratories aerosolize freshly thawed cultures while others grow in liquid broth directly from frozen stocks or first on agar plates followed by culture in liquid broth. Most laboratories utilize MHb for growth and aerosolization of *F. tularensis* although some use CCDM. BHI broth has also been reported to support the growth of *F. tularensis*. In our initial efforts reported here we utilized the LVS strain to infect mice, demonstrating that relative humidity and culture media altered the aerosolization but not the virulence of *F. tularensis*.

Other efforts to evaluate the impact of culture media on *F. tularensis* virulence have been mixed. CCDM was found to enhance virulence of LVS in mice, but only after repeated passage in CCDM in culture which appeared to enhance capsule production (Cherwonogrodzky et al., 1994). In our system, culture in CCDM overnight did not enhance the virulence of LVS, suggesting that some switch/mutation occurred during the multiple passages reported in the prior study that triggered the increased capsule production and virulence. Similar to what was reported by Hazlett et al. for BHI and MHb, we saw no difference in the LD_50_ or time to death between all three culture media (Hazlett et al., 2008). Probit analysis suggests that culturing overnight in BHI did lower the LD_99_, but the number of animals used is not sufficient to establish whether this difference is significant. It is possible that had we used death as an endpoint that some mice that were euthanized might have survived, thereby changing the results of the LD_50/99_ determination. The differences noted between culture conditions in the size of the spleen and bacterial titers in the liver and spleen would support this notion. Evaluation of the clinical signs recorded, however, could argue the opposite as a number of mice that developed clinical signs indicative of severe disease (>10% weight loss, ruffled fur, reduced response to stimuli) did survive.

Our data suggest that there may be a difference in how well the capsule is anchored to the cell membrane based on culture conditions. Hazlett et al. have also reported that BHI-grown LVS behaves more like LVS freshly isolated from macrophages, with a similar mRNA profile. One could hypothesize that perhaps outside of a host cell, *F. tularensis* does not anchor the capsule well to the cell membrane in order to promote shedding of the capsule as a means of immune evasion. Once engulfed by a macrophage, this shedding process might then be deleterious, forcing the bacterium to better anchor the capsule to the membrane. This may have potential implications for the ability of vaccines to protect against *F. tularensis* infection and requires further study.

The observation that spray factor was not only improved but more consistent when using BHI broth was somewhat surprising. It is possible that the fat content, primarily brain lipids, of the broth prevented dehydration of the particle. Data from the desiccation and capsule stripping experiments support the notion that changes in gene expression resulting from culturing in BHI anchored the capsule to the membrane better. The improved anchoring of the capsule would increase resistance to rising salt concentrations in the aerosol particle as the particle reduces in size and desiccates while traveling into the exposure chamber. Results with SCHU S4 were virtually identical indicating that the differences in spray factor were not an LVS or *F. tularensis* type B-only phenomenon.

There is of course concern that reporting the results of experiments such as these might represent “dual-use research” that could be used for nefarious purposes or by terrorists to improve efforts to develop and disseminate biological weapons. In the U.S. government's recently issued policy on dual-use research, it lists specific categories of experiments to consider for potential “dual-use”, including any experiment that “Increases the stability, transmissibility, or the ability to disseminate the agent or toxin” [REF]. Evaluating culture media for the effect on aerosol spray factor could be seen as falling into that category. However, these conclusions cannot be made from this report because spray factor does not assess survival of pathogens in an aerosol and in this case was limited to short duration (10 min) experiments under highly controlled settings in a dynamic chamber. The controlled conditions (e.g., temperature, humidity, light, and air flow rate) achieved in controlled animal exposure chambers are not comparable or reproducible to open systems such as those that pose a bioterrorism concern. Thus, these laboratory-based findings are not directly applicable to intentional dissemination of *F. tularensis* to cause harm. Finally, if these results are not published in the open, peer-reviewed literature then it would be hard to meet the FDA's goal of highly characterized, standardized and reproducible aerosol exposures to meet the requirements of the Animal Rule for license of medical countermeasures against *F. tularensis*.

We report here efforts to establish a standardized method for reproducibly infected animals with aerosols containing *F. tularensis*. The results are in line with prior reports regarding the need to have high relative humidity. Culture media impacted spray factor but did not alter virulence of LVS in mice. Further research is needed to understand the mechanism(s) underlying the differences observed in spray factor resulting from the choice of culture media.

### Conflict of interest statement

The authors declare that the research was conducted in the absence of any commercial or financial relationships that could be construed as a potential conflict of interest.
